# Delayed laparoscopic cholecystectomy for a patient with coronavirus disease 2019 who developed gangrenous cholecystitis: a case report

**DOI:** 10.1186/s40792-022-01494-7

**Published:** 2022-07-18

**Authors:** Yurie Yoshida, Tomohiro Iguchi, Norifumi Iseda, Kosuke Hirose, Takuya Honboh, Noriko Iwasaki, Seiya Kato, Noriaki Sadanaga, Hiroshi Matsuura

**Affiliations:** 1 Department of Surgery, Saiseikai Fukuoka General Hospital, 1-3-46 Tenjin, Chuo-ku, Fukuoka, 810-0001 Japan; 2 Department of Internal Medicine, Saiseikai Fukuoka General Hospital, Fukuoka, Japan; 3 Division of Pathology, Saiseikai Fukuoka General Hospital, 1-3-46 Tenjin Chuo-Ku, Fukuoka, Japan

**Keywords:** COVID-19, Gangrenous cholecystitis, Laparoscopic cholecystectomy

## Abstract

**Background:**

Gangrenous cholecystitis has a high risk of perforation and sepsis; therefore, cholecystectomy in the early stage of the disease is recommended. However, during the novel coronavirus disease 2019 (COVID-19) pandemic, the management of emergent surgeries changed to avoid contagion exposure among medical workers and poor postoperative outcomes.

**Case presentation:**

A 56-year-old man presented to our hospital with abdominal pain. Computed tomography revealed intraluminal membranes, an irregular or absent wall, and an abscess of the gallbladder, indicating acute gangrenous cholecystitis. Early laparoscopic cholecystectomy seemed to be indicated; however, a COVID-19 antigen test was positive despite no obvious pneumonia on chest computed tomography and no symptoms. After discussion among the multidisciplinary team, antibiotic therapy was started and percutaneous transhepatic gallbladder drainage (PTGBD) was planned for the following day because the patient’s vital signs were stable and his abdominal pain was localized. Fortunately, the antibiotic therapy was very effective, and PTGBD was not needed. The cholecystitis improved and the patient was discharged from the hospital on day 10. One month later, laparoscopic delayed cholecystectomy was performed after confirming a negative COVID-19 polymerase chain reaction test result. The postoperative course was uneventful, and the patient was discharged on postoperative day 2 in satisfactory condition.

**Conclusion:**

We have reported a case of acute gangrenous cholecystitis in a patient with asymptomatic COVID-19 disease. This report can help to determine treatment strategies for patients with gangrenous cholecystitis during future pandemics.

## Background

The pathophysiology of gangrenous cholecystitis involves ischemia of the gallbladder wall caused by severe inflammation. Because of the risk of perforation and sepsis [1, 2], cholecystectomy in the early stage of the disease is recommended [3]. After the World Health Organization declared novel coronavirus disease 2019 (COVID-19) to be a pandemic, the management strategies for emergent surgeries changed because of the high risk of contagion exposure among medical workers and the poor postoperative outcomes in patients with COVID-19 [4–6]. COVID-19 has had profound effects on the global healthcare system [7, 8], and the Omicron variant has rapidly spread to numerous countries [9]. Thus, effective management of patients requiring emergency surgery during the COVID-19 pandemic is essential for the safety of both patients and healthcare workers. However, the optimal management strategy remains unclear because of lack of evidence. We herein report a case of gangrenous cholecystitis in a patient with COVID-19 who underwent delayed laparoscopic cholecystectomy following conservative treatment to avoid the risk of environmental contamination, virus exposure, and postoperative morbidity and mortality.

## Case presentation

A 56-year-old man presented to our hospital with a 6-day history of abdominal pain. He had a history of hypertension, diabetes, and *Helicobacter pylori* eradication. Examination revealed a body temperature of 35.6 ℃, pulse of 92 beats/min, blood pressure of 152/90 mmHg, respiratory rate of 18 breaths/min, and oxygen saturation of 98% on room air. Localized pressure pain was found in his right hypochondrium. He had no obvious rebound tenderness. Laboratory data showed an elevated white blood cell count (9.2 × 10^3^/μL) and C-reactive protein concentration (11.81 mg/dL). Liver function tests, including measurement of the alanine aminotransferase, aspartate aminotransferase, and total bilirubin levels, were all within normal limits.

**Fig. 1:**
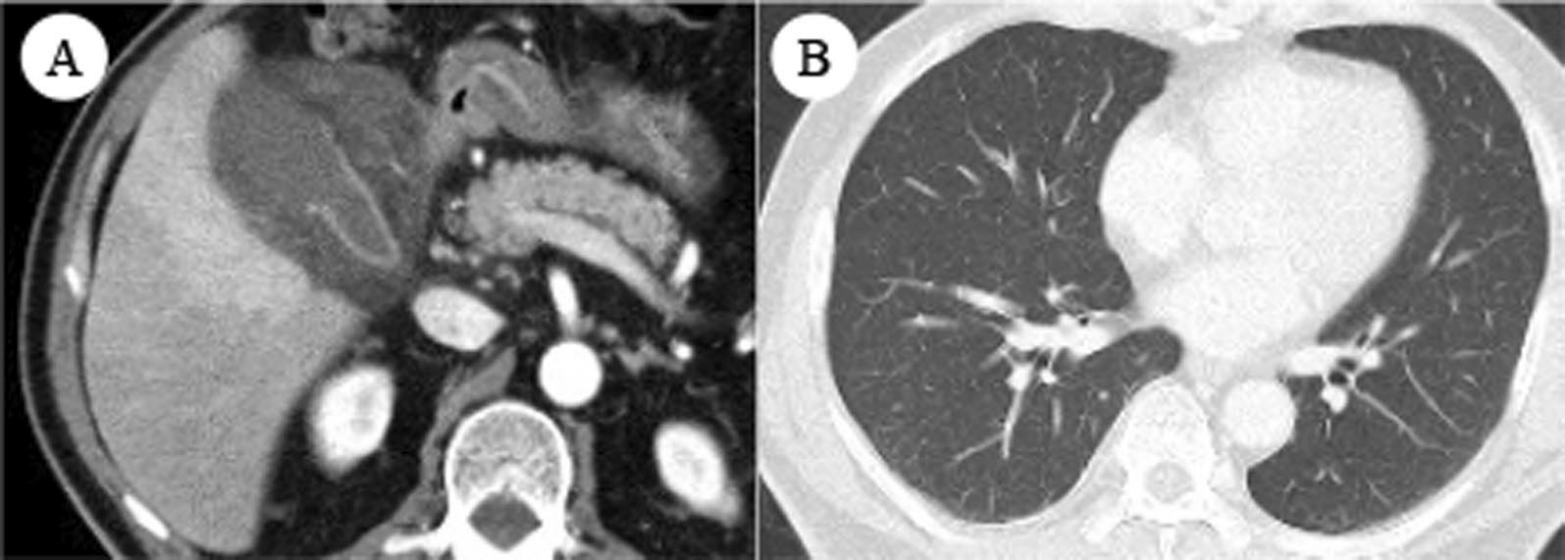
**A** Contrast-enhanced computed tomography revealed specific findings of gangrenous cholecystitis, including intraluminal membranes, an irregular or absent wall, and abscess formation. **B** Chest computed tomography showed no evidence of pneumonia

Enhanced abdominal computed tomography (CT) revealed intraluminal membranes, an irregular or absent wall, and an abscess of the gallbladder. No ascites was present. No gallstones were seen, and the extrahepatic bile duct was normal (Fig. 1). These imaging findings were compatible with the characteristics of acute gangrenous cholecystitis. Although the unvaccinated patient had no history of close contact with a COVID-19 patient and no common clinical symptoms of COVID-19 pneumonia (e.g., fever, cough, dyspnea, or myalgia) and chest CT revealed no obvious pneumonia, a COVID-19 qualitative antigen test using nasopharyngeal swabs for screening was positive at admission. The possibility of false positives was fully considered, however, he should be treated as a COVID-19 infected person, considering contagion and surgical risk. A multidisciplinary team including clinicians from the departments of surgery, internal medicine, anesthesiology, and infectious disease discussed the therapeutic strategy for the patient. Early laparoscopic cholecystectomy seemed to be indicated. However, because of the patient’s asymptomatic COVID-19 infection, it was important to avoid the risk of exposure among healthcare workers and minimize the patient’s perioperative morbidity and mortality. Therefore, antibiotic therapy (sulbactam/cefoperazone) was selected as the initial treatment, and percutaneous transhepatic gallbladder drainage (PTGBD) was planned for the following day because the patient’s vital signs were stable and his abdominal pain was localized. If the gallbladder perforated and presented with pan-peritonitis during the clinical course, we planned to perform emergency laparotomy instead of laparoscopic surgery in order to minimize the operation time after taking infection control measures. Fortunately, the antibiotic therapy was very effective, and PTGBD was not needed. For treatment of COVID-19, the patient was given symptomatic therapy and was quarantined for 10 days. Abdominal CT revealed improvement of the gallbladder inflammation, and the blood test results were also improved. Therefore, the patient was discharged from the hospital on day 10.

**Fig. 2:**
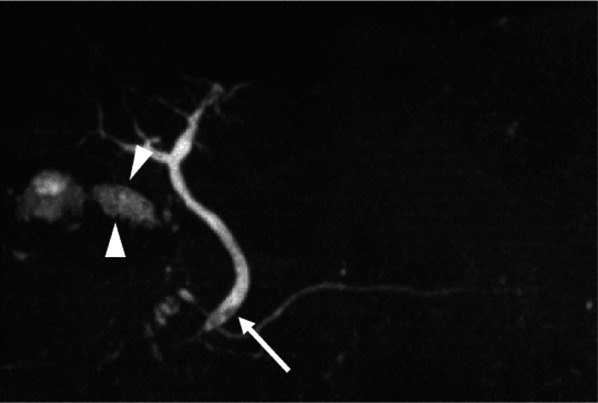
Magnetic resonance cholangiopancreatography revealed small gallstones in the gallbladder (arrowhead) and common bile duct (arrow)

**Fig. 3:**
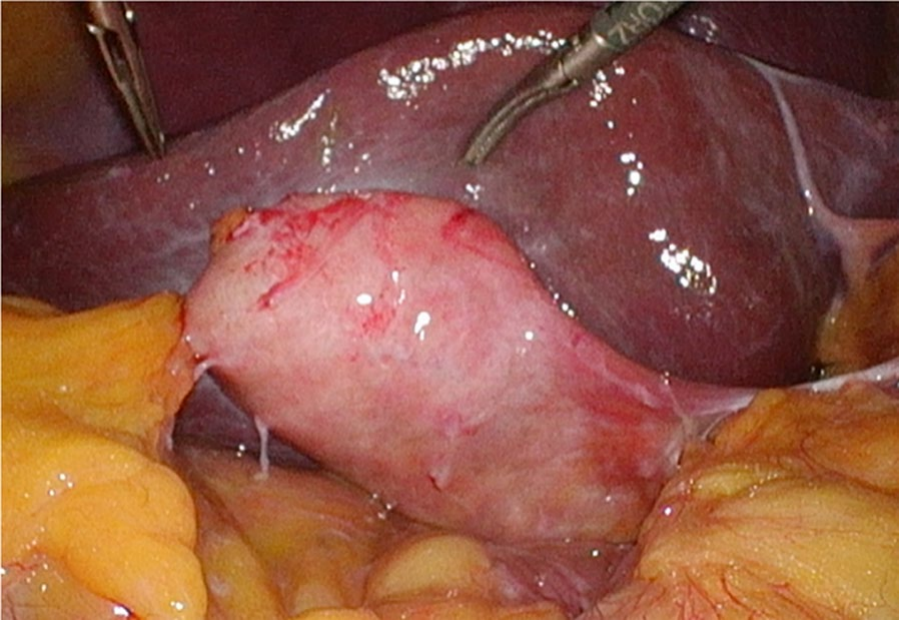
Intraoperative examination revealed a thickened gallbladder wall that was partially inflamed

**Fig. 4:**
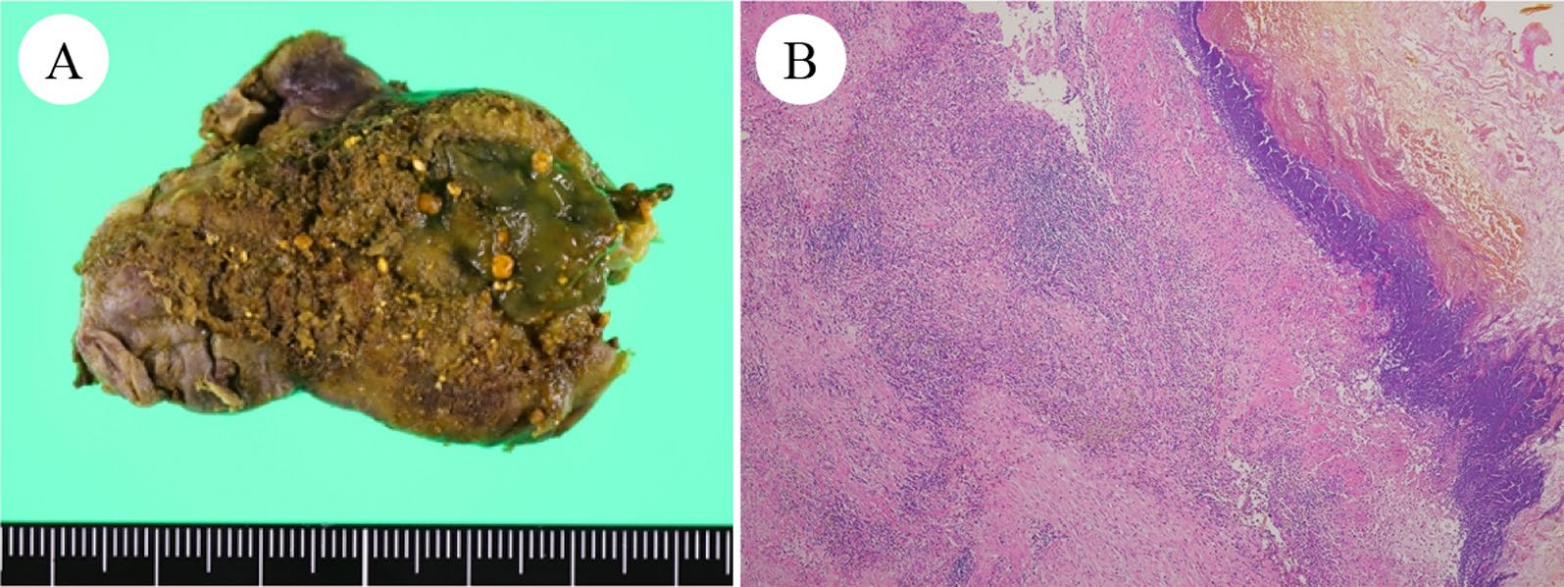
Macroscopically, diffuse mucosal necrosis was observed in the gallbladder fundus (**A**). Microscopic findings showed transmural ischemic necrosis of the gallbladder fundus (**B**)

Considering the high rate of recurrent cholecystitis after conservative management, delayed cholecystectomy was planned. One month later, the patient underwent laparoscopic cholecystitis after confirming a negative COVID-19 polymerase chain reaction test result. Preoperative magnetic resonance cholangiopancreatography revealed filling defects in the gallbladder and common bile duct (Fig. 2). Three days before the surgery, biliary endoscopic sphincterotomy was performed followed by balloon extraction of common bile duct stones. The surgery was carried out under laparoscopy using four ports. Neither ascites nor an abscess was found. However, a thickened gallbladder wall with omental adhesion was observed (Fig. 3). The cystic duct and cystic artery were identified and divided under the critical view of safety, and the cholecystectomy procedure was finished. The operative time was 168 min. Macroscopically, the mucosa of the gallbladder fundus was necrotized (Fig. 4A). Histopathological examination confirmed transmural ischemic necrosis of the gallbladder fundus (Fig. 4B). The postoperative course was uneventful, and the patient was discharged on postoperative day 2 in satisfactory condition.

## Discussion

Gangrenous cholecystitis is a life-threatening disease. It is characterized by necrosis of the gallbladder wall caused by ischemia followed by vascular insufficiency, and it is a risk factor for gallbladder perforation and sepsis in patients with acute cholecystitis [1]. Furthermore, acute perforated cholecystitis is associated with poor outcomes [2]. Hence, according to the Tokyo Guidelines 2018, emergency laparoscopic cholecystectomy is indicated in patients with acute cholecystitis who do not have decreased organ function and an unfavorable performance status score [3]. Early cholecystectomy has been established as the treatment of choice for acute cholecystitis and can be performed as far as 10 days after symptom onset. Early cholecystectomy is associated with a shorter hospital stay, fewer comorbidities, and greater cost-effectiveness [3, 10, 11]. However, during the COVID-19 pandemic, there was an increase in non-surgical treatment to reduce the risk of contagion exposure among medical workers based on a statement by the British Intercollegiate General Surgical Guideline on COVID-19 [12, 13]. This change was implemented because there appeared to be a risk of viral transmission through the aerosols generated by electrocautery and ultrasonic devices used in both open and laparoscopic surgery [14]. The keys to prevention of spread include proper personal protective equipment, proper transport procedures for patients with COVID-19, use of negative-pressure operating rooms, minimal staff, minimal use of electrocautery and high-energy devices, and routine use of smoke evacuation systems.

The safety of cholecystectomy for patients with COVID-19 remains unknown because of the lack of data regarding postoperative outcomes. Doglietto et al. [5] reported a higher rate of respiratory, hemorrhagic, and thrombotic postoperative complications after general anesthesia in patients with COVID-19. Furthermore, another study reviewed 34 patients with asymptomatic COVID-19 undergoing elective surgeries including major surgery. All patients developed COVID-19 pneumonia after surgery, severe complications occurred in 44.1% of the patients and mortality was 20.5% [6]. Thus, cholecystectomy for the patients with asymptomatic COVID-19 seemed to be very high risk compared to non-surgical treatment.

PTGBD has been widely performed for clinically ill patients with acute cholecystitis because of its advantages of minimal invasiveness, a low complication rate, early symptom relief, and improvements in acute inflammation [15]. However, there are some claims that delayed laparoscopic cholecystectomy after PTGBD is more likely to result in conversion to an open procedure because of the development of fibrosis, and this procedure has been associated with higher mortality, a longer hospital stay, more complications, and higher readmission rates [16–18]. In addition, PTGBD may be avoided in patients with gallbladder gangrene. Whereas the delayed operation group who underwent cholecystectomy after treatment including PTGBD for gangrenous cholecystitis had a longer postoperative hospital stay than the early operation group, however, there were no differences between the two groups in surgical factors and postoperative complication [19]. In our case, it corresponded to Grade II (moderate) acute cholecystitis according to the Tokyo Guidelines 2018 [3] and early laparoscopic cholecystectomy was considered to be indicated under a relatively good general condition. However, considering the risks of surgery under general anesthesia for patient with COVID-19, COVID-19 infection had to be regarded as a risk factor for surgery, although it is not included in the guidelines. Thus, PTGBD was scheduled for treatment of the patient’s gangrenous cholecystitis in the negative-pressure room with appropriate personal protective equipment, minimal stuff and minimal time if the conservative treatment resulted in an unfavorable clinical course; fortunately, however, the antibiotic therapy was remarkably effective, and PTGBD was avoided. Nevertheless, PTGBD may be a good option for treatment of gangrenous cholecystitis in patients with COVID-19.

SARS-CoV-2, the pathogen responsible for COVID-19, has a viral spike protein that binds to angiotensin-converting enzyme 2 receptors, which are present in various organs, including the gallbladder; intracellular entry of the virus then occurs [20]. COVID-19-associated cholecystitis caused by such direct vesicular involvement has been described in some case reports [21]. COVID-19 also increases the incidence of systemic endotheliitis, hypercoagulability, and thrombotic microangiopathy, which collectively contribute to the occurrence of cholecystitis [22]. COVID-19 upregulates the expression of proinflammatory cytokines such as interleukin-6 and tumor necrosis factor-alpha, triggering a cytokine storm that recruits macrophages and causes inflammatory reactions [23]. This state of hypercoagulation induced by COVID-19 can lead to gangrenous cholecystitis arising from vascular insufficiency, but this etiology was considered less likely in our case. Instead, acute calculous cholecystitis likely caused the gangrenous cholecystitis in our patient. However, it is possible that the inflammation associated with COVID-19 became a trigger for the gangrenous cholecystitis in this case.

## Conclusions

We have reported a case involving a 56-year-old man with acute gangrenous cholecystitis who was diagnosed with asymptomatic COVID-19 disease at admission. We suggest that delayed laparoscopic cholecystectomy, preceded by PTGBD for patients in whom surgical treatment places them at high risk, may be the best choice for treatment of acute cholecystitis in patients with COVID-19. This report can help to determine treatment strategies for gangrenous cholecystitis during future pandemics.

## Data Availability

The authors declare that all the data in this article are available within the article.

## Abbreviations

COVID-19: Coronavirus disease 2019
PTGBD: Percutaneous transhepatic gallbladder drainage
CT: Computed tomography
SARS-CoV-2: Severe acute respiratory syndrome coronavirus 2
